# The membrane effects of melittin on gastric and colorectal cancer

**DOI:** 10.1371/journal.pone.0224028

**Published:** 2019-10-17

**Authors:** Caroline Soliman, Sarah Eastwood, Vi Khanh Truong, Paul A. Ramsland, Aaron Elbourne

**Affiliations:** 1 School of Science, RMIT University, Bundoora West Campusm Bundoora, Victoria, Australia; 2 Nanobiotechnology Laboratory, RMIT University, Melbourne City Campus, Melbourne, Victoria, Australia; 3 Department of Immunology, Central Clinical School (Monash University), Alfred Medical Research and Education Precinct, Melbourne, Victoria, Australia; 4 Department of Surgery Austin Health (University of Melbourne), Austin Health, Heidelberg, Victoria, Australia; University of PECS Medical School, HUNGARY

## Abstract

The cytotoxic effects of melittin, a bee-venom peptide, have been widely studied towards cancer cells. Typically, these studies have examined the effect of melittin over extended-time courses (6–24 hours), meaning that immediate cellular interactions have been overlooked. In this work, we demonstrate the rapid effects of melittin on both gastric and colorectal cancer, specifically AGS, COLO205 and HCT-15 cell lines, over a period of 15 minutes. Melittin exhibited a dose dependent effect at 4 hours of treatment, with complete cellular death occurring at the highest dose of 20 μg/mL. Interestingly, when observed at shorter time points, melittin induced cellular changes within seconds; membrane damage was observed as swelling, breakage or blebbing. High-resolution imaging revealed treated cells to be compromised, showing clear change in cellular morphology. After 1 minute of melittin treatment, membrane changes were observed, and intracellular material could be seen expelled from the cells. Overall, these results enhance our understanding of the fast acting anti-cancer effects of melittin.

## Introduction

Cancer is a leading cause of mortality worldwide, currently accounting for approximately 1 in 6 deaths [1]. A recent report by the World Health Organisation (WHO) estimated that in 2018 18 million cases of cancer were diagnosed, and 9.6 million cancer related deaths occurred. Colorectal and gastric cancers are the third and fifth most commonly diagnosed cancers, accounting for 10% and 6% of cancer diagnoses, respectively. As such, it is unsurprising that these cancer types are responsible for high mortality rates, largely due to their poor prognosis [1]. Currently, cancer therapies consist mainly of surgical intervention, chemo- or radio-therapy, and gene or hormone therapy. Unfortunately, there is still a distinct lack of targeted treatments available despite recent developments, including antibody therapeutics, peptides and other small molecule therapeutics [2–4].

Melittin is a widely studied cytolytic peptide derived from bee venom and is considered a model for both cationic and other cytolytic peptides. Interestingly, it displays broad spectrum efficacy as an anti-viral, anti-bacterial, anti-fungal, anti-parasitic and anti-tumour agent [2, 5–7]. This is because the cytolytic actions of melittin are non-selective, affecting both signal transduction and regulatory pathways. As such, melittin induces multiple cell death mechanisms, including apoptosis, inhibition of proliferation or angiogenesis, cell cycle arrest, and inhibition of cancer motility, migration, metastasis and invasion. For cancer treatment, the cytolytic activity of melittin has been examined on a variety of cell types over recent years [8–15]. During apoptosis, cell lysis is induced via phospholipid bilayer disruption, pore formation and inducing permeability [6]. For gastric cancer cells, melittin has been shown to induce dose and time-dependant apoptosis and necrosis, inhibiting the proliferation of AGS cells. These affects were visualised as cell shrinkage, cell shape irregularity, cellular detachment and membrane damage [11]. Moreover, colon cancer cell lines (HCT-116, CT26 and LS174T) have only been tested with melittin conjugates [6]. Melittin also induced apoptosis through mitochondrial pathways in SGC-7901 gastric cancer cells [10]. In addition, when comparing melittin sensitivity of cancer cells to normal cells, one study showed that melittin was significantly more cytotoxic to human lung cancer cells than to the control human lung fibroblasts cells [16].

Although melittin is known to kill cancer cells by inducing apoptosis, visualisation studies have been somewhat limited. Recent atomic force microscopy (AFM) studies, performed on lipid monolayers, revealed distinct morphological changes and clear pore formation after the addition of melittin [17]. However, whole-cell studies have predominantly investigated the effect of melittin at long time points (6–24 hours), meaning that immediate effects have been poorly described. Despite extensive study, developing melittin as a therapeutic agent for cancer treatment remains challenging, mainly due to its non-specific cellular lytic activity, as well as its short lifetime in the blood and potential to cause severe toxic reactions upon intravenous injection [6]. The most serious side effect of melittin is due to its haemolytic activity, which is its ability to lyse red blood cells [18]. Studies have shown that melittin binds tightly to human red blood cells, resulting in channels large enough for haemoglobin leakage and ultimately cell lysis, with 50% lysis occurring at only 10% occupancy of melittin binding sites [18, 19]. However, more recent research has focussed towards melittin conjugates and derivatives as alternates for use in combination and targeted cancer therapies. Furthermore, immuno-conjugation, nanotechnology and gene therapy are being used to develop melittin-based therapies with increased specificity and selectivity and reduced toxicity and limit off-target cytolysis [5, 20–25].

In this study, we examine the rapid effect of melittin treatments on gastric and colorectal cancer cells. Specifically, the cancer cell lines AGS, COLO205 and HCT-15 were investigated as model systems to observe the effects of melittin at short time points. Firstly, the dosage dependence of melittin on cancer cell death was examined within the concentration range of 0.5–20 μg/mL. For all cancer types investigated, the effective concentration range was between 5–10 μg/mL. At low concentrations (0.5–5 μg/mL) of melittin, no significant effect on viability was observed. At higher concentrations, all samples displayed significant degrees of melittin induced cytotoxicity, with almost complete cellular inactivation at doses of 20 μg/mL. Following this, high-resolution imaging was conducted at short time points at a dose of 10–20 μg/mL to observe the cellular changes that occur upon immediate addition of melittin. A combination of scanning electron microscopy (SEM), AFM, and time-lapsed confocal laser scanning microscopy (CLSM) was employed to gain a more complete understanding of the membrane changes that occur when melittin exhorts its cytotoxic effects on cancer cells. Knowledge of the fast kinetics of cytolysis is an important step in developing targeted melittin-based cancer therapy.

## Methods

### Cell culture

Human adenocarcinoma cell lines AGS, COLO205 and HCT-15 were obtained from Cell Bank Australia. Cell lines were authenticated using short tandem repeat profiling and quality controlled using Biotool Mycoplasma Detection Kit-QuickTest. Cells were cultured in 25cm^2^ and 75cm^2^ tissue culture flasks under sterile conditions using complete RPMI 1640 media with L-glutamine (containing 10% Fetal Bovine Serum (FBS) and 1% Penicillin-Streptomycin) at 37 ^o^C with 5% CO_2_. Cells were split when 70% confluent using 0.25% Trypsin-EDTA and cultured to a maximum of 15 passages. Cells were harvested periodically in freeze media (FBS with 10% DMSO) for storage at -80 ^o^C and for long-term storage in liquid nitrogen.

### Flow cytometry (FACS)

Cells were seeded at 1 × 10^5^ cells per well in a 96-well plate and treated with melittin at 0.5–20 μg/mL in complete RPMI media for 4 hours at 37 ^o^C with 5% CO_2_. The positive control was treated with 0.1% Triton X-100 in PBS for 15 minutes. Cells were washed and then resuspended in 1 μg/mL of propidium iodide (PI) solution in PBS for up to 5 minutes before being analysed in the FACS Canto (10,000 cells per sample). The resultant data was analysed by FlowJo software where cell populations were gated by cell size and complexity to exclude doublets, following which PI positive populations (PE emission >10^3^) were selected. Two-way ANOVA tests were used to assess statistical significance in cell death between melittin concentrations for each cell line. Data was graphed as mean ± standard deviation with n = 4.

### Optical microscopy

Glass coverslips were coated with poly-D-lysine at 1 mg/mL and cells were seeded overnight in complete RPMI media. Cells were examined by optical microscopy (Nikon Eclipse TS100 microscope) to visualise changes that occur upon the addition of 10 μg/mL or 20 μg/mL melittin diluted in PBS over a 15-minute time course.

### Live/Dead staining and florescence microscopy

Glass coverslips were coated with poly-D-lysine at 1 mg/mL and cells were seeded overnight in complete RPMI media. Cells were treated with melittin at 0.5–20 μg/mL for either 1 minute, 15 minutes or 4 hours at 37 ^o^C with 5% CO_2_. Samples were then incubated with a solution of 4 μM Ethidium homodimer (EthD-1) and 2 μM Calcein-AM in PBS for 45 minutes at room temperature (Invitrogen). Coverslips were mounted onto slides and examined by fluorescence microscopy for red (dead) and green (live) fluorescence. Samples were examined using a Leica DM2500 epifluorescence microscope with a DFC310 digital camera, and images were captured using LAS software (V4.1; Leica Microsystems).

### Membrane staining and confocal imaging

Glass coverslips were coated with poly-D-lysine at 1 mg/mL and cells were seeded overnight in complete RPMI media. Samples were incubated with 2 μg/mL of Dil (1,1'-dioctadecyl-3,3,3',3'-tetramethylindocarbocyanine perchlorate) membrane dye in PBS for 30 minutes in the dark at 37 ^o^C with 5% CO_2_. Cells were examined by confocal microscopy (Olympus FV1200 microscope) to visualise changes that occur upon the addition of 20 μg/mL melittin diluted in PBS over a 15-minute time course. Images shown are representative of cells in each sample.

### AFM sample preparation

Glass coverslips were coated with poly-D-lysine at 1 mg/mL and cells were seeded overnight in complete RPMI media. Cells were treated with melittin for 1 minute at 20 μg/mL in PBS and then fixed in either 2.5% glutaraldehyde or 8% formaldehyde.

### AFM imaging

All cells were imaged in PBS to examine morphology. Each system was studied using a MFP-3D Bio (Oxford Instruments, Asylum Research, Santa Barbara, CA, USA) at room temperature (25°C) using amplitude modulated-AFM (AM-AFM). BL-TR400PB cantilevers (Oxford Instruments, Asylum Research, Santa Barbara, CA, USA, nominal spring constant kc = 0.09 N/m). To minimize the imaging force, a setpoint ratio (Imaging Amplitude (A)/free amplitude (A0)) of >0.7–0.8 was maintained, which has been shown to minimize any tip-sample distortion and damage for a variety of materials [26–28]. Each cantilever was calibrated using the thermal spectrum method prior to use and the lever sensitivity was determined using force spectroscopy; the spring constant was resolved via the inverse optical lever sensitivity (InVOLS) using force curve measurements on the hard glass surface prior to fluid immersion.

### AFM image analysis

AFM data was processed using a combination of the Asylum research software, custom MATLAB codes, and Gwyddion software [29]. No image filtering (noise deconvolution, scar removal, or Fast Fourier Transform (FFT) filtering) was applied.

### SEM

Silicon wafers were pre-coated with poly-D-lysine at 1 mg/mL and cells were seeded overnight in complete RPMI media before being treated with melittin for 1 minute in PBS. Prior to SEM imaging, all samples were fixed using 3% glutaraldehyde, dehydrated with series of ethanol (20%, 50%, 70%, 90%, 100%), and finally coated with a thin film of gold (10 nm). Scanning electron micrographs were obtained using a field-emission scanning electron microscope (FE-SEM). A FEI Verios 460L FEGSEM (FEI company, Oregon, United States) at 5 kV was used. Some images were post-modified for brightness and contrast using Adobe Photoshop.

## Results

### Cancer cell death induced by melittin as a function of concentration

**Fig 1:**
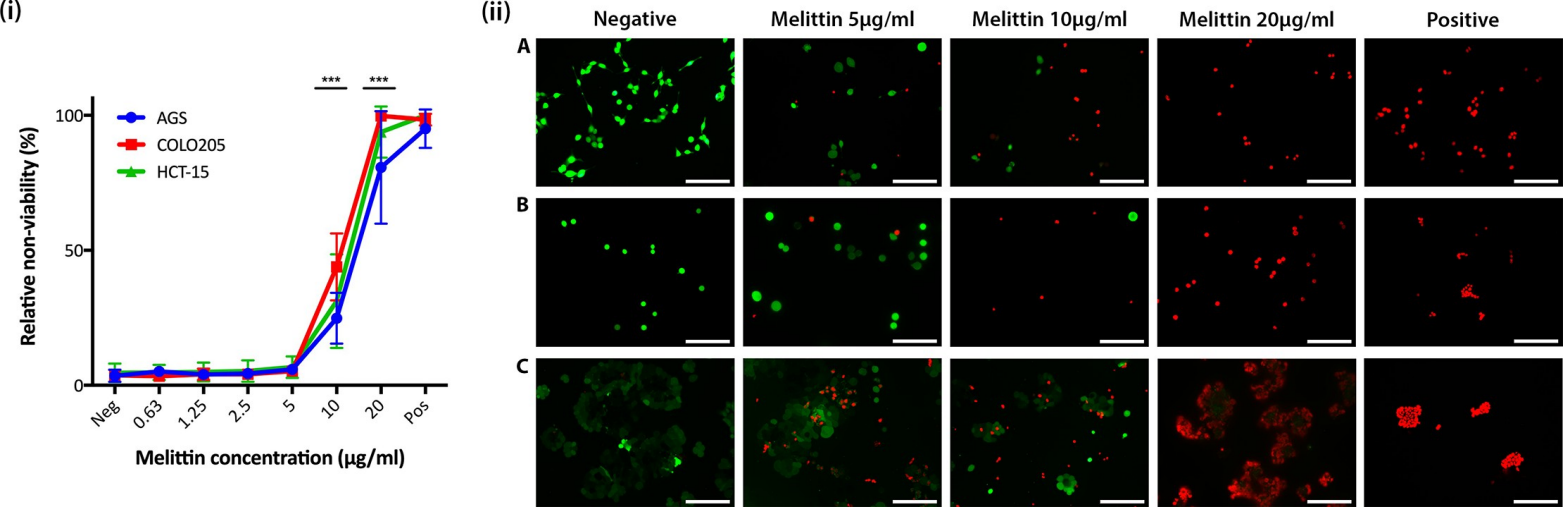
**(i)** Propidium iodide (PI) uptake showing death of gastric and colorectal cancer cell lines following a 4-hour melittin treatment. Cells were treated with 0.5–20 μg/mL melittin and the positive control was treated with 0.1% Triton X-100. Data shown as mean ± SD where n = 4. *** represents p = <0.001. EC50 values are 14 μg/mL for AGS, 11 μg/mL for COLO205 and 13 μg/mL for HCT-15. **(ii)** Florescence images showing Live/Dead staining of gastric and colorectal cancer cell lines following a 4-hour melittin treatment. **A)** AGS, **B)** COLO205 and **C)** HCT-15 cells were grown on poly-D-lysine coated coverslips overnight and treated with melittin at 5 μg/mL, 10 μg/mL and 20 μg/mL for 4 hours. The positive control was treated with Triton X-100. All cells were stained with Calcein-AM live stain (green) and EthD-1 dead stain (red). Average viability was determined for AGS (78% for 5 μg/mL, 17% for 10 μg/mL and 0% for 20 μg/mL), COLO205 (86% for 5 μg/mL, 40% for 10 μg/mL and 0% for 20 μg/mL), and HCT-15 (73% for 5 μg/mL, 50% for 10 μg/mL and 4% for 20 μg/mL). Viability for all negative and positive controls was 99–100% and 0% respectively. Scale bars represent 100 microns.

To identify the concentration range at which melittin causes cancer cell death, cytotoxicity was measured through propidium iodide (PI) uptake by non-viable cells and analysed by FACS. Fig 1 shows the relative non-viability of the cancer cell lines AGS, COLO205, and HCT-15 as a function of melittin concentration. Inspection of the data reveals that melittin inhibits the growth of both gastric and colorectal cancer types (gastric line AGS and colorectal lines COLO205 and HCT-15) in a dose dependent manner (Fig 1(i)). As expected, the number of non-viable cells increased as melittin concentration increased. No significant decrease in viability was seen in the concentration range of 0.5–5 μg/mL of melittin (Fig 1(i)). Assessment of cellular viability as a function of melittin concentration revealed that the effective concentration range for all cancer types was between 5–20 μg/mL, with 24.8 ± 9.4%, 31.2 ± 17% and 43.9 ± 12.4% cell death seen for AGS, HCT-15 and COLO205 respectively for the 10μg/mL treatment (P = 0.0009). Compared to untreated controls, all samples treated with 20 μg/mL of melittin displayed significant death (P = 0.0009). At a melittin concentration of 20 μg/mL, AGS showed the lowest cell death, with 80.6 ± 20% cell death, whereas HCT-15 and COLO205 showed similar percentages at 93.8 ± 9.6% and 99.7 ± 0.17% respectively. Positive control samples showed approximately a 95% decrease in viability for cell lines treated with 0.1% Triton X-100 in PBS. No plateau phase was reached for the 4-hour treatment (Fig 1(i)), and a similar reduction in cell viability was seen following 12-hours of treatment (data not shown). In general, non-viable cells within the populations readily uptake PI dye, and are visually seen to reduce in both cell size and internal complexity, indicating cytotoxicity. Comparatively, viable cell populations are not stained by PI and exhibited higher cellular size [30, 31]. This allowed meaningful comparison between live and dead cells to be achieved.

While PI is a useful marker for cellular death, it cannot be used to detect the precise cytotoxic effects induced by melittin exposure. To overcome this experimental limitation, a Live/Dead cell staining kit was employed to directly measure the proportion of live vs. dead cells at melittin concentrations of 5 μg/mL, 10 μg/mL and 20 μg/mL (Fig 1(ii)), following 4 hours of exposure. CLSM micrographs show the corresponding fluorescence images obtained following staining for the cancer cell lines AGS, COLO205, and HCT-15, as well as the respective controls (Fig 1(ii)A–1C). For all cell lines, cytotoxic activity of melittin follows a significant dose dependent killing, with the proportion of dead cells (red EthD-1 stained) increasing dramatically compared to live (green calcein-AM stained) as the concentration of melittin increased (Fig 1(ii)). Melittin effects were compared to non-treated samples, showing 100% viability, and Triton X-100 treated samples showing 100% cell death in all cell lines.

Interestingly, AGS treated with 5 μg/mL and 10 μg/mL melittin displayed a noticeable shift in cytotoxicity, with more than 50% of the cell population stained red at 10 μg/mL (Fig 1(ii)A). For COLO205 it was difficult to measure proportions of live and dead cells due to the semi-adherent nature of this cell line, although a dramatic decrease in live cells was observed at 10 μg/mL melittin (Fig 1(ii)B). High levels of cell death were observed for HCT-15 at 5μg/mL melittin, and this proportion did not significantly differ at 10 μg/mL (Fig 1(ii)C). At 20 μg/mL melittin, AGS and COLO205 showed 100% cell death. However, due to the cell clumping nature of HCT-15, cells situated in the centre of the clumps show some viability, whilst cells along the outer edge of the clumps were dead, resulting in approximately 95% cell death (Fig 1(ii)C). Together, these results suggest the cytolytic effects of melittin vary somewhat as a function of cell line, as well as cellular adherence and aggregation. Importantly, the results obtained by live and dead cell staining were commensurate with those obtained by FACS (Fig 1).

### Melittin causes rapid changes to cell membranes

**Fig 2:**
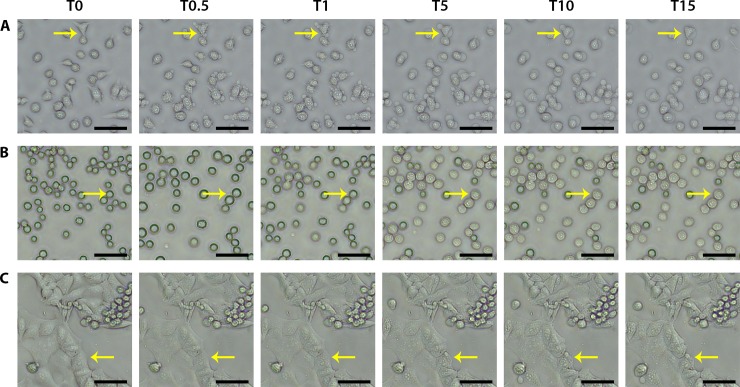
Optical microscopy images showing membrane swelling, granulation and blebbing of gastric and colorectal cancer cells after the addition of melittin. **A)** AGS, **B)** COLO205 and **C)** HCT-15 cells were grown on poly-D-lysine coated coverslips overnight and imaged in PBS using a light microscope before (T0) and after (T0.5, 30 seconds; T1, 1 minute; T5, 5 minutes; T10, 10 minutes; T15, 15 minutes) the addition of 20 μg/mL melittin. After melittin addition, the effects on the cells can be seen almost immediately. The yellow arrow points to membrane changes. Scale bars represent 50 microns.

Melittin (20 μg/mL) was added to each cancer cell line and observed by optical microscopy to elucidate the effects of melittin as a function of time. The highest dose was chosen to elicit an observable response. Prior to imaging the gastric cancer cells (AGS) showed normal morphology, which was indicated by smooth membrane edges, intact nuclei and regular fusiform shaping (Fig 2A). Similar morphologies were observed for all other cancer cell lines, COLO205 and HCT-15, however with the added adherence and aggregation inherent to each cell type, as described above (Fig 2B and 2C, respectively). The initial morphology of each cell line serves as a baseline for visualising cellular changes induced by the introduction of melittin. Interestingly, very rapid changes were observed for AGS at the cell membrane upon the addition of melittin (Fig 2). Within 30 seconds of the addition of melittin, blebbing can be seen, where small bulges begin to occur at the periphery of the cells. Over the 15-minute time course, this process continues with changes noticed in the membrane at each time point. In addition, cell-surface detachment occurred following membrane changes (Fig 2A, S1 Movie).

Different effects were observed on the colorectal cancer cell lines tested, with COLO205 cells appearing as regular spherical cells with smooth surfaces. Cells showed swelling at the 30 second to 1 minute time points, followed by granulation in the presence of melittin. Due to the semi-adherent nature of this cell line, cells begin to move in solution almost immediately after the addition of melittin, and very few cells remain adhered after 15 minutes of treatment (Fig 2B, S2 Movie). Comparatively, HCT-15 cells formed large adherent clumps and maintained its aggregate behaviour during treatment. Only subtle changes can be seen over the 15-minute time course, mostly affecting the cells located at the aggregate edges; cells in these positions were observed to shrink and become rougher in the presence of melittin (Fig 2C, S3 Movie). This observation is consistent with the fluorescence images obtained during differential Live/Dead staining (Fig 1C).

### Melittin treatment results in cell death within 15 minutes

**Fig 3:**
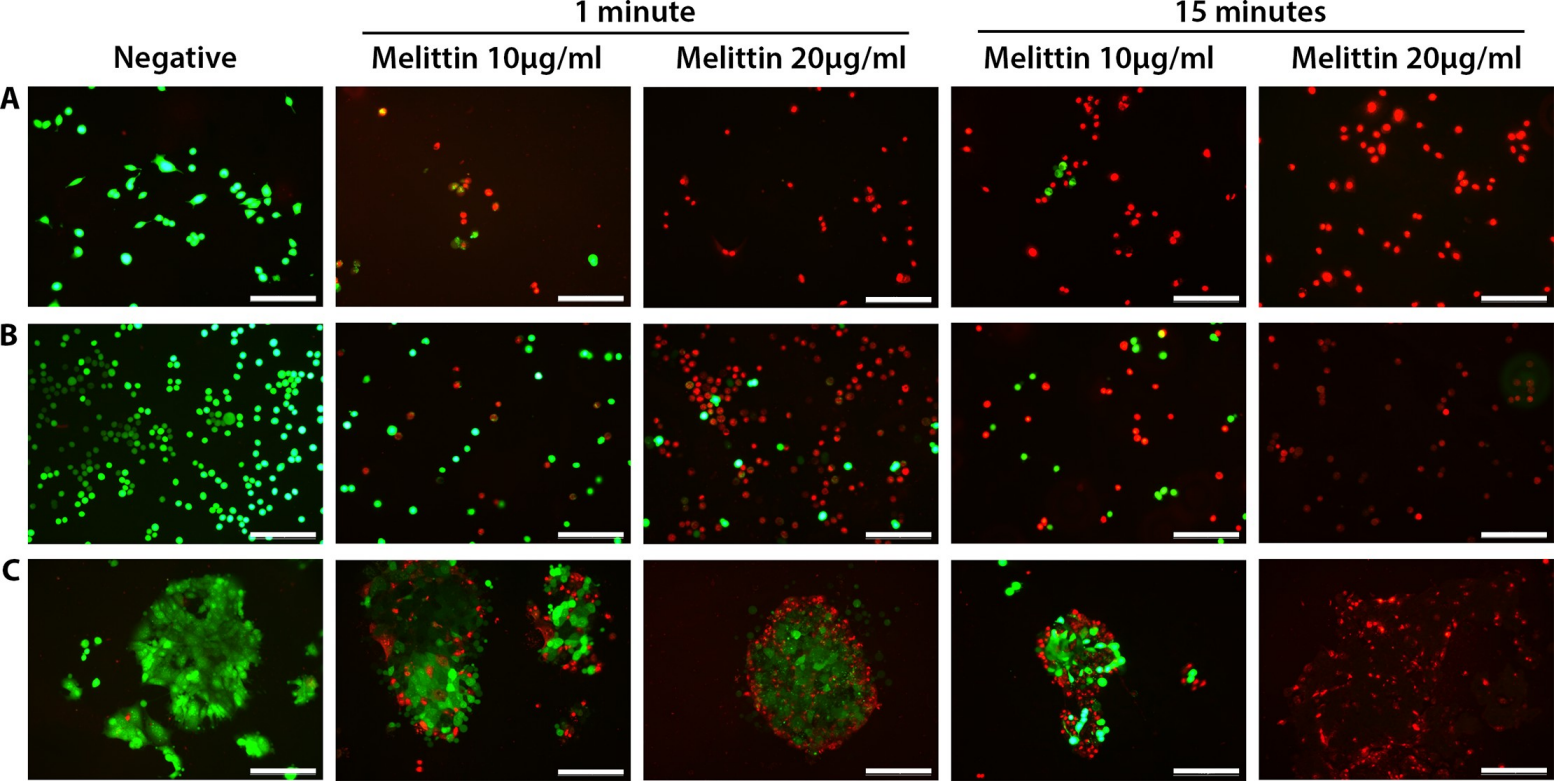
Florescence images showing Live/Dead staining of gastric and colorectal cancer cell lines following short melittin treatments. **A)** AGS, **B)** COLO205 and **C)** HCT-15 cells were grown on poly-D-lysine coated coverslips overnight and treated with melittin at 10 μg/mL or 20 μg/mL for either 1 minute or 15 minutes. All cells were stained with Calcein-AM live stain (green) and EthD-1 dead stain (red). Average viability was determined after 1 minute for AGS (53% for 10 μg/mL and 1% for 20 μg/mL), COLO205 (59% for 10 μg/mL and 12% for 20 μg/mL), and HCT-15 (62% for 10 μg/mL and 51% for 20 μg/mL), and after 15 minutes for AGS (3% for 10 μg/mL and 0% for 20 μg/mL), COLO205 (38% for 10 μg/mL and 3% for 20 μg/mL), and HCT-15 (34% for 10 μg/mL and 0% for 20 μg/mL). Viability for all negative controls was 96–99%. Scale bars represent 100 microns.

To determine if the membrane changes observed under optical microscopy resulted in cell death, Live/Dead staining was performed on cancer cells at shorter treatment times. Gastric (AGS) and colorectal cells (COLO205 and HCT-15) were treated with melittin at both 10 μg/mL and 20 μg/mL for 1 and 15 minutes, respectively (Fig 3). Prior to treatment, all cells displayed regular, smooth morphologies with >95% viability as expected within a natural population (Fig 3). This data provides a baseline for post-treatment analysis. Overall, as the treatment concentration and time increased, so did cell death, with complete cell death seen at the higher dose after 15 minutes for all cell lines (Fig 3). At these shorter time frames, melittin appeared to exhibit its highest killing efficiency on AGS, with over 50% of cells staining red (dead) at 10 μg/mL after 1 minute, and 100% death observed at 20 μg/mL after 1 minute. Complete death was also seen for the higher concentration at 15 minutes (Fig 3A). For colorectal lines COLO205 and HCT-15, only a small percentage of cells were killed using the 10 μg/mL 1 minute treatment, and this number increased for the 20 μg/mL sample. After 15 minutes of 10 μg/mL treatment, some live cells are still observed (<25%) (Fig 3B and 3C). For HCT-15 it should be noted that cell death at 1 minute begins to occur for cells mostly located at the surrounding edges of the cellular clumps. As time progresses, the killing effect is then observed for cells located more centrally in the clumps (Fig 3C), likely showing a diffusion-based cell lysis mechanism.

### Membrane alterations

**Fig 4:**
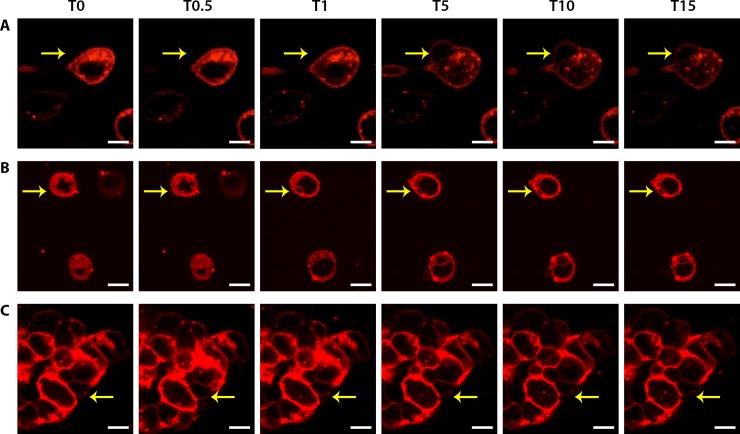
Membrane changes before and after melittin treatment at different time points for **A)** AGS, **B)** COLO205 and **C)** HCT-15. After the addition of melittin, cells undergo membrane swelling followed by membrane blebbing. Cells were grown on poly-D-lysine coated coverslips overnight and stained with DIL membrane dye (red) and imaged by confocal microscopy before (T0) and after (T0.5, 30 seconds; T1, 1 minute; T5, 5 minutes; T10, 10 minutes; T15, 15 minutes) the addition of 20 μg/mL melittin. The yellow arrow points to membrane changes. Scale bars represent 10 microns.

To confirm the membrane effect observed thus far, a membrane dye (1,1'-Dioctadecyl-3,3,3',3'-Tetramethylindocarbocyanine Perchlorate–Dil) was utilised to examine the changes occurring after the introduction of melittin. Cells were stained, and then observed by confocal laser scanning microscopy (CLSM) while introducing high-doses of melittin (20 μg/mL). Fig 4 shows representative time-lapse *in situ* CLSM images of the three cell lines prior to and following treatment (Fig 4, S4–S6 Movies). Importantly, different effects were observed for all cell lines. For AGS, the cell showed an elongated morphology with a long cellular attachment, typical of their cell type [32]. Upon the addition of melittin, the cell began to swell slightly, following which a large membrane bleb (see yellow arrow, Fig 4A) developed and then continued to grow over the time course. The morphology of the attachment itself also changed via swelling followed by shrinkage and possible detachment from the surface. In addition, the surface of the AGS cell itself appeared to become more granulated by the 15 minute time point (Fig 4A, S4 Movie), commensurate with the optical microscopy (Fig 2) and Live/Dead florescence images (Fig 3).

Similar effects were observed for COLO205, where the cells can be seen swelling, following which multiple small membrane blebs begin to appear (Fig 4B, S5 Movie). In comparison to AGS (Fig 4A), these blebs are a fraction of the size but appear to cover a large proportion of the COLO205 cell membrane (Fig 4B). For HCT-15, the effects of melittin are subtler; the cell aggregate initially showed swelling at 30 seconds and then began to shrink over 15 minutes. This can be seen through thinning of the stained region around the periphery indicating cytoplasmic shrinkage, although at an individual cell level only hints of cell membrane damage are observed (Fig 4C, S6 Movie).

### High-resolution imaging of membrane and cellular damage caused by melittin treatment

**Fig 5:**
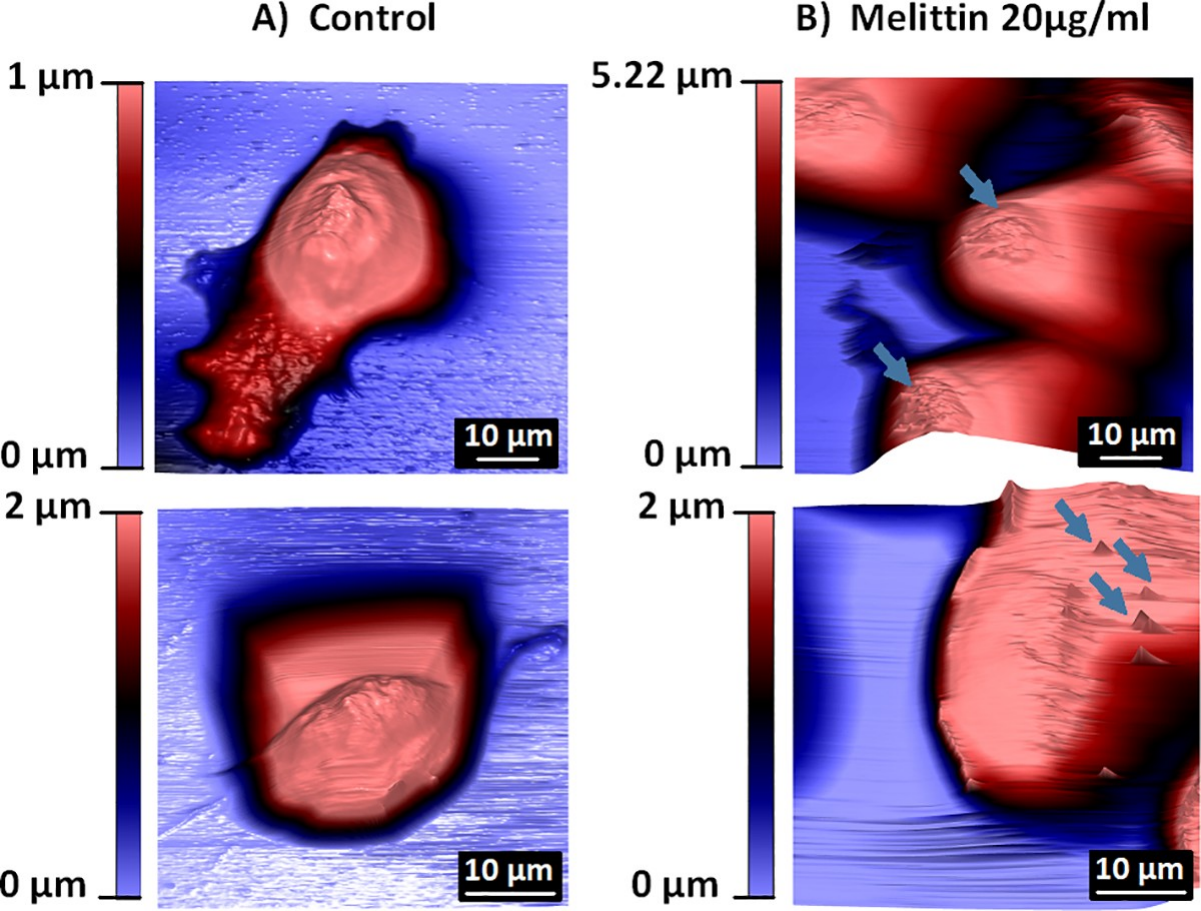
AFM images showing the morphology of AGS cells **A)** under normal conditions (no treatment) and **B)** after 20 μg/mL melittin treatment. AGS cells were grown on poly-D-lysine coated coverslips overnight, treated with 20 μg/mL melittin treatment for 1 minute and then fixed in 2.5% gluteraldehyde or 8% formaldehyde. Cells were imaged in PBS on the Asylum MFP-3D. Blue arrows indicate cellular damage. Scale bars are as indicated in the respective images.

While the methods used have extensively demonstrated that cellular death is occurring through membrane changes, mostly membrane blebbing, high-resolution imaging techniques were needed to assess these effects in detail. AFM was performed on AGS cells to better characterise the observed morphological changes. Firstly, untreated AGS cells were imaged as a baseline, where multiple cell morphologies were observed (Fig 5). All cells were intact and had clear nuclear regions, with either a spherical shape or an elongated shape, and different cellular attachments were also observed. After high-dose (20 μg/mL), short-duration melittin treatment, the AGS cell membrane appeared to become ‘sticky’ (blue arrows) as indicated by the streaky nature of the corresponding AFM images. This ‘sticky’ quality most likely corresponds to the loss of membrane integrity described thus far and confirms that cellular damage is occurring almost immediately after treatment (Fig 5). AFM characterisation was not performed on the colorectal cell lines COLO205 and HCT-15 because of their respective semi-adherent and clumping natures making them challenging to image by this technique. However, SEM was undertaken to confirm rapid membrane changes.

**Fig 6:**
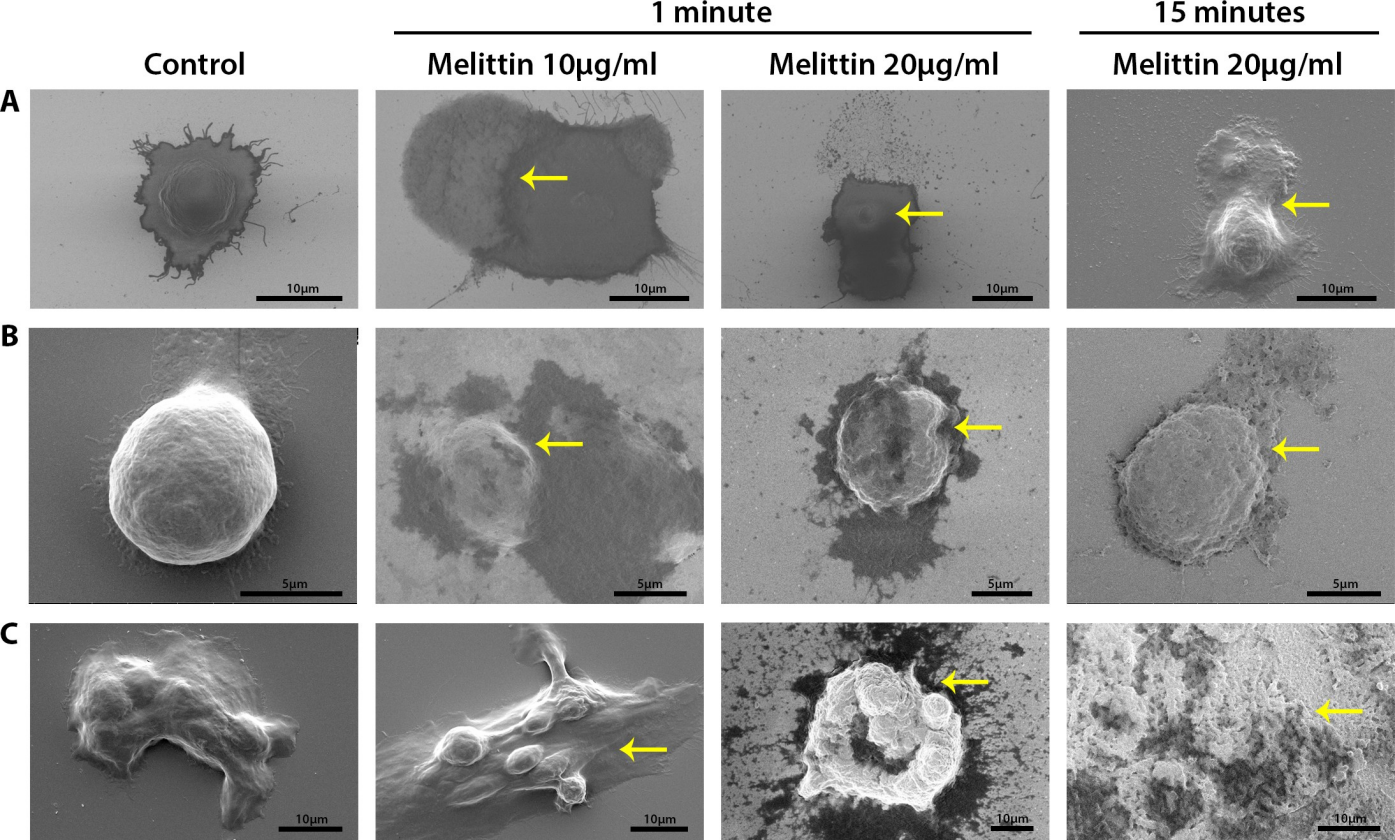
SEM images of cancer cells showing differences in cellular morphology before and after melittin treatment (10 μg/mL and 20 μg/mL) for **A)** AGS, **B)** COLO205 and **C)** HCT-15. Cells were grown on poly-D-lysine coated silicon chips overnight and treated with melittin for 1 minute or 15 minutes. Yellow arrows point to cellular and membrane damage. Scale bars are as indicated in the respective images.

SEM imaging was performed on all cell lines to visualise the morphology of cancer cells without/with melittin treatment. Prior to treatment, SEM images showed typical cell morphology of AGS, COLO205 and HCT-15 with visible nuclear regions and no clear membrane damage (Fig 6 control). After melittin treatment, AGS cell morphology changed due to a loss of membrane integrity. At 10 μg/mL melittin, intracellular matter was seen leaking out of an AGS cell (Fig 6A). At 20 μg/mL melittin, there is a large protrusion in the membrane and the top section of the cellular membrane appears to have broken open to release intracellular matter across the surface (Fig 6A). Comparatively, the release of intracellular matter for COLO205 occurs in a different manner. At both 10 μg/mL and 20 μg/mL melittin treatments, the nuclear region is no longer clearly visible, and the cell membrane appears more granular and has buckled in an irregular manner. Small visible pores were observed across the membrane surface, indicating a loss of cellular membrane integrity (Fig 6B). For HCT-15, cellular damage is not as clearly visible at the lower concentration, but there is noticeable cellular damage at the higher concentration. At baseline, clumps of cells are irregular in shape and formation. After melittin treatment, additional cellular matter is visible on the surface, indicating a loss of membrane integrity with some visible membrane damage (Fig 6C). By 15 minutes of melittin treatment, colorectal cancer cells were completely damaged, with damaged cells and small granular matter remaining on the surface.

## Discussion

A wide body of literature has shown that melittin induces cellular death via a non-specific lytic mechanism, which has prompted its study as a potential anti-viral, anti-bacterial, anti-fungal, anti-parasitic and anti-tumour treatment. In addition, melittin is known to be a cationic, pore forming peptide [6, 33]. With regard to cancer, the effects of melittin on cancer cells and lipid (bi)-layers have been examined by an array of methods over extended periods of time (i.e. 6 to 24 hours) within concentration ranges of 0.5 to 10 μg/mL [8–15, 17, 34]. However, the cytolytic effects at shorter time frames have been largely overlooked.

Here, melittin was observed to produce a dose dependent cell lysis effect against AGS, COLO205, and HCT-15 cell lines, a finding commensurate with several studies in the field. Some of these studies were performed on cancer cells using melittin or melittin conjugates [8–15], while others looked at melittin on lipid layers [17, 34]. However, in our study the dose needed to render all cells inactivated was somewhat higher, specifically more than double, than that of concentrations needed against other cancer cell lines [9–11, 14, 15]. In addition, different effects were observed on each cell line examined, although a dose of 20 μg/mL of melittin resulted in complete cell death for all systems studied. Together these findings may indicate that previous studies are not a general indication of the effect of melittin against all cancer cell lines, meaning that melittin activity may be cell line specific.

Importantly, our results demonstrate the rapid effect of melittin on gastric and colorectal cancer cells. Melittin induces cell membrane damage within 1 minute of treatment, where granulation, blebbing and cell swelling followed by shrinkage were observed (Figs 2–6), with complete cell death occurring within 15 minutes (Fig 3). Previously, the shortest duration study observed melittin on DPPC monolayers for 4 hours [17], while the shortest time-point studies looked at melittin treatment on gastric cancer cells and human lymphoblastoid cells after 1 hour of incubation [10, 35]. With regard to gastric cancer, a previous study observed less than 15% viability for AGS cells treated with 8 μg/mL of melittin that had been extracted from Iranian bees after 6–24 hours of treatment [11]. To our knowledge, no studies have been conducted on the colorectal cell lines tested here, although some studies have looked at the effects of melittin conjugates on other colorectal cells [6]. Understanding the immediate membrane damage caused by melittin provides essential information for developing targeted melittin therapeutics.

Membrane effects have been well studied in cancer using cancer cells and lipid layers by different methods and at longer treatment times, often looking at pore formation as well as membrane alteration including perturbation and membrane disruption [11, 14, 25, 34, 36–38]. A number of studies have also examined the mechanistic effects of melittin looking at pathways leading to apoptosis and cell death, including induction of death receptors [5, 6, 9, 10, 37, 39, 40]. Broadly, it is thought that melittin disrupts lipid membranes via the toroidal and detergent mechanisms, depending on the membrane lipid to peptide ratio [41]. At lower concentrations, melittin induces small or transient pores whereby cellular contents do not seep from the cell, but transmembrane ions reorientate, altering membrane charge [38, 42]. Transient pores occur after the α-helical melittin peptide monomers interact with the cell surface, dimerize and shallowly penetrate the membrane [43, 44]. Upon melittin aggregation, larger, more stable pores are formed and transmembrane leakage occurs at concentrations in the micromolar range [43–45]. At concentrations of peptide exceeding lipid ratios, melittin can disintegrate membranes [46]. These findings are commensurate with the findings of this study, where some level of cell death is noted from 5 μg/mL, suggesting transient pores occur at lower concentrations of melittin. While the complete lysis of cells was observed at the highest melittin dose for all cell lines (Fig 1). In addition, variable pore size induced by melittin can be explained by structural changes upon interactions with the bilayers or interactions between peptide monomers. While pore formation was not observed here at shorter time frames, several other damaging membrane effects were observed, almost immediately after the introduction of melittin (Figs 2–4). SEM imaging revealed the formation of pores in membrane surfaces for some cells after treatment (Fig 6). In agreement with our findings, a previous study has also demonstrated vesicle formation and loss of membrane integrity via electron microscopy following short duration, very high dose melittin treatment (2 minutes of 200 μg/mL) [35]. However, this study was performed in human lymphoblastoid cells, and limited work has been done in solid tumours.

Interestingly, the effects of melittin appear to be altered on the aggregating cell line investigated. AGS and COLO205 cells appear much more sensitive to melittin when compared to HCT-15, with very noticeable membrane changes affecting all treated cells in the samples (Figs 2–5). However, for HCT-15, which forms cell clumps, melittin first kills cells located at the periphery and then slowly diffuses into the centre of the clumps to ultimately result in cell death in a dose and time dependent manner. In this regard, the cell clumps of HCT-15 act like solid tumours. It has been demonstrated that drug and antibody penetration of solid tumours is problematic for a number of reasons relating to the tumour microenvironment, including elevated pressure and large vascular systems, as well as mechanisms that can lead to resistance, such as drug export pumps and changes in metabolic pathways [47, 48]. These findings give some insight into the effect melittin may have on solid tumours and could in part influence development of delivery methods for melittin or similar cytolytic peptides to tumours for cancer therapy.

Despite extensive studies, developing melittin as a therapeutic agent for cancer treatment remains challenging, mainly due to its non-specific cellular lytic activity. This includes its ability to lyse red blood cells [6, 18]. In an attempt to overcome this, melittin research has focussed on the development on melittin conjugates and derivatives. Examples include melittin attached by cleavable linkers [23], environment-sensitive peptide delivery systems [20] and a number of melittin based fusion proteins [5, 21, 22, 24, 25]. In addition, through the development of novel linker strategies, melittin-loaded nanoparticles and nanocarriers have also been studied for delivering melittin to the tumour microenvironment [49–51]. Despite these caveats regarding implementation, understanding the underlying mechanism of action is imperative for the development of next-generation melittin-based therapies. Importantly, melittin has proven to be toxic to cancer cells and has the potential to cause tumour death. Developing alternative methods for peptide delivery will allow for novel cancer therapeutics, with increased specificity and selectivity and considerably reduced toxicity by limiting off-target cytolysis. Knowledge of the fast kinetics of cytolysis is an important step towards realising the goal of a targeted melittin-based cancer therapy.

## Conclusion

While the wide-reaching cytotoxic effects of melittin have been well documented, its instantaneous effects on cancer cells have not in the past been examined. Here, we demonstrated the clear membrane effects of high dose melittin on gastric and colorectal cancer cells over a 15-minute time course, with cellular changes occurring within seconds in the form of cell swelling, membrane blebbing and breakage. All observed effects were dose and time dependent, with a higher dosage needed in comparison to previous studies. Additionally, variation in membrane damage occurred between different cell lines, with the aggregating cell line demonstrating a clear diffusion effect mimicking that of a solid tumour. The findings of this study enhance our knowledge of anti-cancer peptides by giving an insight into the rapid effects of the model peptide melittin on cancer cells.

## Supporting information

S1 Movie Optical microscopic movie revealing the changes induced by melittin for AGS cells as a function of time. (MP4)

S2 Movie Optical microscopic movie revealing the changes induced by melittin for COLO205 cells as a function of time. (MP4)

S3 Movie Optical microscopic movie revealing the changes induced by melittin for HCT-15 cells as a function of time. (MP4)

S4 Movie Movie of time-lapse CLSM images showing the membrane alteration of AGS cells. (MP4)

S5 Movie Movie of time-lapse CLSM images showing the membrane alteration of COLO205 cells. (MP4)

S6 Movie Movie of time-lapse CLSM images showing the membrane alteration of HCT-15 cells. (MP4)

## Abbreviations

AFM: atomic force microscopy
CLSM: confocal laser scanning microscopy
Dil: 1,1'-dioctadecyl-3,3,3',3'-tetramethylindocarbocyanine perchlorate
DMSO: dimethyl sulfoxide
DPPC: dipalmitoylphosphatidylcholine
EthD-1: ethidium homodimer
FBS: fetal bovine serum
FE-SEM: field-emission scanning electron microscope
PBS: phosphate buffered saline
PI: propidium iodide
SEM: scanning electron microscopy
WHO: world health organisation

## References

[1] Latest global cancer data: Cancer burden rises to 18.1 million new cases and 9.6 million cancer deaths in 2018 [press release]. 12 September 2018 2018.

[2] Wang S, Jia M. Antibody Therapies in Cancer In: Zhang S, editor. Progress in Cancer Immunotherapy. Dordrecht: Springer Netherlands; 2016 p. 1–67.

[3] Yavari B, Mahjub R, Saidijam M, Raigani M, Soleimani M. The Potential Use of Peptides in Cancer Treatment. Curr Protein Peptide Sci. 2018;19(8):759–70. 29332577 10.2174/1389203719666180111150008

[4] Marqus S, Pirogova E, Piva TJ. Evaluation of the use of therapeutic peptides for cancer treatment. J Biomed Sci. 2017;24(1):21–36. 10.1186/s12929-017-0328-x 28320393 PMC5359827

[5] Liu C-c, Hao D-j, Zhang Q, An J, Zhao J-j, Chen B, et al Application of bee venom and its main constituent melittin for cancer treatment. Cancer Chemother Pharmacol. 2016;78(6):1113–30. 10.1007/s00280-016-3160-1 27677623

[6] Rady I, Siddiqui I, Rady M, Mukhtar H. Melittin, a major peptide component of bee venom, and its conjugates in cancer therapy. Cancer Lett. 2017;402:16–31. 10.1016/j.canlet.2017.05.010 28536009 PMC5682937

[7] Premratanachai P, Chanchao C. Review of the anticancer activities of bee products. Asian Pacific Journal of Tropical Biomedicine. 2014;4(5):337–44. 10.12980/APJTB.4.2014C1262 25182716 PMC3985046

[8] Choi JH, Jang AY, Lin S, Lim S, Kim D, Park K, et al Melittin, a honeybee venom-derived antimicrobial peptide, may target methicillin-resistant Staphylococcus aureus. Mol Med Report. 2015;12(5):6483–90. 10.3892/mmr.2015.4275 PMC4626175 26330195

[9] Jo M, Park MH, Kollipara PS, An BJ, Song HS, Han SB, et al Anti-cancer effect of bee venom toxin and melittin in ovarian cancer cells through induction of death receptors and inhibition of JAK2/STAT3 pathway. Toxicol Appl Pharmacol. 2012;258(1):72–81. 10.1016/j.taap.2011.10.009 22027265

[10] Kong G-M, Tao W-H, Diao Y-L, Fang P-H, Wang J-J, Bo P, et al Melittin induces human gastric cancer cell apoptosis via activation of mitochondrial pathway. World J Gastroenterol. 2016;22(11):3186–95. 10.3748/wjg.v22.i11.3186 27003995 PMC4789993

[11] Mahmoodzadeh A, Zarrinnahad H, Bagheri KP, Moradia A, Shahbazzadeh D. First report on the isolation of melittin from Iranian honey bee venom and evaluation of its toxicity on gastric cancer AGS cells. J Chin Med Assoc. 2015;78(10):574–83. 10.1016/j.jcma.2015.06.008 26316200

[12] Mularski A, Wilksch JJ, Wang H, Hossain MA, Wade JD, Separovic F, et al Atomic Force Microscopy Reveals the Mechanobiology of Lytic Peptide Action on Bacteria. Langmuir: the ACS journal of surfaces and colloids. 2015;31(22):6164–71. 25978768 10.1021/acs.langmuir.5b01011

[13] Rajabnejad SH, Mokhtarzadeh A, Abnous K, Taghdisi SM, Ramezani M, Razavi BM. Targeted delivery of melittin to cancer cells by AS1411 anti-nucleolin aptamer. Drug Dev Ind Pharm. 2018;44(6):982–7. 10.1080/03639045.2018.1427760 29325460

[14] Zarrinnahad H, Mahmoodzadeh A, Hamidi M, Mahdavi M, Moradi A, Bagheri K, et al Apoptotic Effect of Melittin Purified from Iranian Honey Bee Venom on Human Cervical Cancer HeLa Cell Line. Int J Pept Res Ther. 2018;24(4):563–70. 10.1007/s10989-017-9641-1 30416405 PMC6208649

[15] Zhang S-F, Chen Z. Melittin exerts an antitumor effect on non-small cell lung cancer cells. Mol Med Report. 2017;16(3):3581–6. 10.3892/mmr.2017.6970 28713976

[16] Tipgomut C, Wongprommoon A, Takeo E, Ittiudomrak T, Puthong S, Chanchao C. Melittin Induced G1 Cell Cycle Arrest and Apoptosis in Chago-K1 Human Bronchogenic Carcinoma Cells and Inhibited the Differentiation of THP-1 Cells into Tumour- Associated Macrophages. Asian Pac J Cancer Prev. 2018;19(12):3427–34. 10.31557/APJCP.2018.19.12.3427 30583665 PMC6428562

[17] Giménez D, Sánchez-Muñoz OL, Salgado J. Direct observation of nanometer-scale pores of melittin in supported lipid monolayers. Langmuir: the ACS journal of surfaces and colloids. 2015;31(10):3146–58. 25705986 10.1021/la504293q

[18] Tosteson M, Holmes S, Razin M, Tosteson D. Melittin lysis of red cells. An International Journal for Studies on the Structure, Function, and Genesis of Biomembranes. 1985;87(1):35–44. 10.1007/BF01870697 4057243

[19] DeGrado WF, Musso GF, Lieber M, Kaiser ET, Kezdy FJ. Kinetics and mechanism of hemolysis induced by melittin and by a synthetic melittin analogue. Biophys J. 1982;37(1):329–38. 10.1016/S0006-3495(82)84681-X 7055625 PMC1329147

[20] Bei C, Bindu T, Remant KC, Peisheng X. Dual secured nano-melittin for the safe and effective eradication of cancer cells. J Mater Chem B. 2014;3(1):25–9. 10.1039/C4TB01401D 25734006 PMC4342614

[21] Ling CQ, Li B, Zhang C, Zhu DZ, Huang XQ, Gu W, et al Inhibitory effect of recombinant adenovirus carrying melittin gene on hepatocellular carcinoma. Ann Oncol. 2005;16(1):109–15. 10.1093/annonc/mdi019 15598947

[22] Qian C-Y, Wang K-L, Fang F-F, Gu W, Huang F, Wang F-Z, et al Triple-controlled oncolytic adenovirus expressing melittin to exert inhibitory efficacy on hepatocellular carcinoma. Int J Clin Exp Pathol. 2015;8(9):10403 26617748 PMC4637563

[23] Sun D, Sun M, Zhu W, Wang Z, Li Y, Ma J. The anti-cancer potency and mechanism of a novel tumor-activated fused toxin, DLM. Toxins (Basel). 2015;7(2):423 10.3390/toxins7020423 25658509 PMC4344633

[24] Wang D, Hu L, Su M, Wang J, Xu T. Preparation and functional characterization of human vascular endothelial growth factor-melittin fusion protein with analysis of the antitumor activity in vitro and in vivo. Int J Oncol. 2015;47(3):1160 10.3892/ijo.2015.3078 26166416

[25] Zhao H, Feng X, Han W, Diao Y, Han D, Tian X, et al Enhanced binding to and killing of hepatocellular carcinoma cells in vitro by melittin when linked with a novel targeting peptide screened from phage display. Journal of Peptide Science. 2013;19(10):639–50. 10.1002/psc.2542 24014474

[26] Dufrêne YF, Ando T, Garcia R, Alsteens D, Martinez-Martin D, Engel A, et al Imaging modes of atomic force microscopy for application in molecular and cell biology. Nature Nanotechnology. 2017;12(4):295–307. 10.1038/nnano.2017.45 28383040

[27] Müller DJ, Dufrêne YF. Atomic force microscopy: a nanoscopic window on the cell surface. Trends Cell Biol. 2011;21(8):461–9. 10.1016/j.tcb.2011.04.008 21664134

[28] Parot P, Dufrêne YF, Hinterdorfer P, Le Grimellec C, Navajas D, Pellequer J-L, et al Past, present and future of atomic force microscopy in life sciences and medicine. J Mol Recognit. 2007;20(6):418–31. 10.1002/jmr.857 18080995

[29] Nečas D, Klapetek P. Gwyddion: an open-source software for SPM data analysis. Central European Journal of Physics. 2012;10(1):181–8.

[30] Elmore S. Apoptosis: a review of programmed cell death. Toxicol Pathol. 2007;35(4):495–516. 10.1080/01926230701320337 17562483 PMC2117903

[31] Wlodkowic D, Skommer J, Darzynkiewicz Z. Flow cytometry-based apoptosis detection. Methods Mol Biol. 2009;559:19–32. 10.1007/978-1-60327-017-5_2 19609746 PMC3863590

[32] Barranco SC, Townsend CM Jr., Casartelli C, Macik BG, Burger NL, Boerwinkle WR, et al Establishment and characterization of an in vitro model system for human adenocarcinoma of the stomach. Cancer Res. 1983;43(4):1703–9. 6831414

[33] Walsh EG, Maher S, Devocelle M, O'Brien PJ, Baird AW, Brayden DJ. High content analysis to determine cytotoxicity of the antimicrobial peptide, melittin and selected structural analogs. Peptides. 2011;32(8):1764–73. 10.1016/j.peptides.2011.06.006 21703316

[34] Lee M-T, Sun T-L, Hung W-C, Huang HW. Process of inducing pores in membranes by melittin. Proc Natl Acad Sci USA. 2013;110(35):14243 10.1073/pnas.1307010110 23940362 PMC3761581

[35] Weston KM, Alsalami M, Raison RL. Cell membrane changes induced by the cytolytic peptide, melittin, are detectable by 90 degrees laser scatter. Cytometry. 1994;15(2):141–7. 10.1002/cyto.990150207 8168400

[36] Dempsey CE. The actions of melittin on membranes. BBA—Reviews on Biomembranes. 1990;1031(2):143–61. 10.1016/0304-4157(90)90006-x 2187536

[37] Oršolić N. Bee venom in cancer therapy. Cancer Metastasis Rev. 2012;31(1):173–94. 22109081 10.1007/s10555-011-9339-3

[38] Tosteson MT, Tosteson DC. The sting. Melittin forms channels in lipid bilayers. Biophys J. 1981;36(1):109–16. 10.1016/S0006-3495(81)84719-4 6269667 PMC1327579

[39] Gajski G, Garaj-Vrhovac V. Melittin: A lytic peptide with anticancer properties. Environ Toxicol Pharmacol. 2013;36(2):697–705. 10.1016/j.etap.2013.06.009 23892471

[40] Son DJ, Lee JW, Lee YH, Song HS, Lee CK, Hong JT. Therapeutic application of anti-arthritis, pain-releasing, and anti-cancer effects of bee venom and its constituent compounds. Pharmacol Ther. 2007;115(2):246–70. 10.1016/j.pharmthera.2007.04.004 17555825

[41] Mihajlovic M, Lazaridis T. Antimicrobial peptides in toroidal and cylindrical pores. BBA—Biomembranes. 2010;1798(8):1485–93. 10.1016/j.bbamem.2010.04.004 20403332 PMC2885466

[42] Hanke W, Methfessel C, Wilmsen H-U, Katz E, Jung G, Boheim G. Melittin and a chemically modified trichotoxin form alamethicin-type multi-state pores. BBA—Biomembranes. 1983;727(1):108–14. 10.1016/0005-2736(83)90374-7 6824646

[43] Liu J, Xiao S, Li J, Yuan B, Yang K, Ma Y. Molecular details on the intermediate states of melittin action on a cell membrane. BBA—Biomembranes. 2018;1860(11):2234–41. 10.1016/j.bbamem.2018.09.007 30409519

[44] Ramirez L, Shekhtman A, Pande J. Nuclear Magnetic Resonance-Based Structural Characterization and Backbone Dynamics of Recombinant Bee Venom Melittin. Biochemistry. 2018;57(19):2775 10.1021/acs.biochem.8b00156 29668274 PMC6333091

[45] Sun D, Forsman J, Woodward CE. Multistep Molecular Dynamics Simulations Identify the Highly Cooperative Activity of Melittin in Recognizing and Stabilizing Membrane Pores. Langmuir: the ACS journal of surfaces and colloids. 2015;31(34):9388–401. 26267389 10.1021/acs.langmuir.5b01995

[46] Ladokhin AS, Selsted ME, White SH. Sizing membrane pores in lipid vesicles by leakage of co-encapsulated markers: pore formation by melittin. Biophys J. 1997;72(4):1762–6. 10.1016/S0006-3495(97)78822-2 9083680 PMC1184370

[47] Khawar IA, Kim JH, Kuh H-J. Improving drug delivery to solid tumors: Priming the tumor microenvironment. J Controlled Release. 2015;201:78–89. 10.1016/j.jconrel.2014.12.018 25526702

[48] Tannock IF, Lee CM, Tunggal JK, Cowan DSM, Egorin MJ. Limited Penetration of Anticancer Drugs through Tumor Tissue. Clin Cancer Res. 2002;8(3):878–84. 11895922

[49] Hou KK, Pan H, Ratner L, Schlesinger PH, Wickline SA. Mechanisms of Nanoparticle-Mediated siRNA Transfection by Melittin-Derived Peptides. ACS Nano. 2013;7(10):8605–15. 10.1021/nn403311c 24053333 PMC4013830

[50] Pan H, Soman NR, Schlesinger PH, Lanza GM, Wickline SA. Cytolytic peptide nanoparticles (‘NanoBees’) for cancer therapy. Wiley Interdisciplinary Reviews: Nanomedicine and Nanobiotechnology. 2011;3(3):318–27. 10.1002/wnan.126 21225660

[51] Soman NR, Baldwin SL, Hu G, Marsh JN, Lanza GM, Heuser JE, et al Molecularly targeted nanocarriers deliver the cytolytic peptide melittin specifically to tumor cells in mice, reducing tumor growth. The Journal of Clinical Investigation. 2009;119(9):2830–42. 10.1172/JCI38842 19726870 PMC2735896

